# Elements Modulating the Prion Species Barrier and Its Passage Consequences

**DOI:** 10.1371/journal.pone.0089722

**Published:** 2014-03-07

**Authors:** Juan-Maria Torres, Juan-Carlos Espinosa, Patricia Aguilar-Calvo, María-Eugenia Herva, Aroa Relaño-Ginés, Ana Villa-Diaz, Mónica Morales, Beatriz Parra, Elia Alamillo, Alejandro Brun, Joaquín Castilla, Susana Molina, Steve A. C. Hawkins, Olivier Andreoletti

**Affiliations:** 1 Centro de Investigación en Sanidad Animal (CISA-INIA), Valdeolmos, Madrid, Spain; 2 CIC bioGUNE and IKERBASQUE, Basque Foundation for Science, Derio-Bilbao, Bizkaia, Spain; 3 Animal Health and Veterinary Laboratories Agency (AHVLA) New Haw, Addlestone, Surrey, United Kingdom; 4 UMR INRA-ENVT 1225, Interactions Hôte Agent Pathogène, École Nationale Vétérinaire de Toulouse, France; INRA, France

## Abstract

The specific characteristics of Transmissible Spongiform Encephalopathy (TSE) strains may be altered during passage across a species barrier. In this study we investigated the biochemical and biological characteristics of Bovine Spongiform Encephalopathy (BSE) after transmission in both natural host species (cattle, sheep, pigs and mice) and in transgenic mice overexpressing the corresponding cellular prion protein (PrP^C^) in comparison with other non-BSE related prions from the same species. After these passages, most features of the BSE agent remained unchanged. BSE-derived agents only showed slight modifications in the biochemical properties of the accumulated PrP^Sc^, which were demonstrated to be reversible upon re-inoculation into transgenic mice expressing bovine-PrP^C^. Transmission experiments in transgenic mice expressing bovine, porcine or human-PrP revealed that all BSE-derived agents were transmitted with no or a weak transmission barrier. In contrast, a high species barrier was observed for the non-BSE related prions that harboured an identical PrP amino acid sequence, supporting the theory that the prion transmission barrier is modulated by strain properties (presumably conformation-dependent) rather than by PrP amino acid sequence differences between host and donor.

As identical results were observed with prions propagated either in natural hosts or in transgenic mouse models, we postulate that the species barrier and its passage consequences are uniquely governed by the host PrP^C^ sequence and not influenced by other host genetic factors. The results presented herein reinforce the idea that the BSE agent is highly promiscuous, infecting other species, maintaining its properties in the new species, and even increasing its capabilities to jump to other species including humans. These data are essential for the development of an accurate risk assessment for BSE.

## Introduction

Bovine spongiform encephalopathy (BSE) in cattle, like scrapie in sheep and goats, is a transmissible spongiform encephalopathy (TSE). The key event in TSE infection is the conversion of the normal cellular prion protein (PrP^C^, which is encoded by the *prnp* gene) into an abnormal disease-associated isoform (PrP^Sc^) in tissues of infected individuals. Conversion of PrP^C^ into PrP^Sc^ is a post-translational process involving structural modifications of the protein and resulting in a higher β-sheet content [1]. PrP^C^ is completely degraded after controlled digestion with proteinase K (PK) in the presence of detergents. In contrast, PrP^Sc^ is N-terminally truncated under such conditions, resulting in a PK resistant core, termed PrP^res^ [2]. PrP^res^, also named PrP 27–30, is a disease marker for TSE and the presence of PrP^Sc^ seems to associate with infectivity [2], [3]. According to the “protein only” hypothesis, PrP^Sc^ represents the infectious TSE agent itself [4].

The outcome of the inoculation of a TSE isolate derived from one species into a different species is unpredictable. In a number of cases, no TSE will develop. In other cases, although TSE will not develop clinically, there might be silent/subclinical infection. If transmission occurs, the TSE agent can either remain identical to the original or evolve into a completely different prion strain [5]–[10]. This phenomenon is named the transmission barrier and despite intensive investigation it remains largely enigmatic. The TSE transmission barrier seems to be the result of a complex interaction between the TSE agent and certain host factors [11], [12].

BSE agent has demonstrated particular capacities to cross species barriers. Natural BSE transmission to humans, with the emergence of variant Creutzfeldt-Jakob disease (vCJD) [13], [14], to cats, to a range of zoo animals [15], and likely to goat [16] has occurred. Meanwhile, experimental transmissions to numerous other species like sheep, pigs, bank voles, mice, and non-human primates [17]–[20] have also been reported. The possibility that BSE infection might occur in other species has important implications for public health.

Interestingly, after passage in different host species, the BSE agent conserved its strain specific signature as assessed by bioassay in RIII mice [21]. More recently, we reported that the BSE agent derived from sheep exhibited an increased transmission efficiency over the original cattle-BSE isolate when assessed in a transgenic mouse model expressing either bovine, porcine or human-PrP [22]–[24]. Other authors have also confirmed that BSE passage through sheep increase its transmission efficiency in a different model of human-PrP transgenic mice [25]. This observation raised crucial questions concerning the BSE agent after passage in species other than bovine.

In this study we investigated the biochemical and biological characteristics of BSE after transmission in both natural host species (cattle, sheep, pigs, mice and humans) and in transgenic mice overexpressing the corresponding PrP^C^ amino acid sequences. The properties of these BSE-derived prions were characterized in comparison with other non-BSE related prions with the aim of gaining a better understanding of the species barrier phenomenon and the consequences of passage across a species barrier.

## Materials and Methods

### Ethics statement

Animal experiments were carried out in strict accordance with the recommendations in the guidelines of the Code for Methods and Welfare Considerations in Behavioural Research with Animals (Directive 86/609EC) and all efforts were made to minimize suffering. Experiments were approved by the Committee on the Ethics of Animal Experiments (CEEA) of the Spanish *Instituto Nacional de Investigación y Tecnología Agraría y Alimentaria* (INIA); Permit Number: CEEA2012/024 and CEEA2009/004.

### Sources and generation of the inocula

*Cattle-BSE* material was obtained from the brainstem of one BSE-affected cow (RQ 225:PG817/00), and supplied by the Animal Health and Veterinary Laboratories Agency (AHVLA), New Haw, Addlestone, Surrey, UK.

*BoTg-BSE* was obtained from the brain of terminally ill BSE-inoculated transgenic mice expressing bovine PrP (BoPrP-Tg110) [26].

*Sheep-BSE* was obtained from the brain of a terminally ill ARQ/ARQ sheep intracerebrally inoculated with BSE, and provided by the French National Institute for Agricultural Research (INRA), Nouzilly, France.

*OvTg-BSE* was obtained from the brain of terminally diseased BSE-inoculated transgenic mice expressing ARQ-ovine PrP (Tg IX) [27].

*Pig-BSE* was obtained from the brain of one BSE-affected pig (PG 33/03) intracerebrally inoculated with BSE as previously described [19], and supplied by the Animal Health and Veterinary Laboratories Agency (AHVLA) New Haw, Addlestone, Surrey, UK.

*PoTg-BSE* was obtained from the brain of terminally diseased BSE-inoculated transgenic mice expressing porcine PrP (PoPrP-Tg001) [28].

*Mouse-BSE* was obtained from the brain of terminally diseased BSE-inoculated C57/Bl6 mice.

*MoTga20-BSE* was obtained from the brain of terminally diseased BSE-inoculated transgenic mice expressing mouse PrP (Tga20) [29].

*Human-vCJD* (met 129) isolate (Ref.: NHBY0/0014) was obtained from the National Institute for Biological Standards and Control, Potters Bar, United Kingdom.

*HuTg-BSE* was obtained from the brain of terminally diseased BSE-inoculated transgenic mice expressing M129-Human PrP (HuPrP-Tg340) [24].

*Atypical Cattle-BSE H* isolate was obtained from the brainstem of one naturally affected cow, diagnosed as atypical H-type BSE (French case 03-2095), and provided by the *Agence Française de Sécurité Sanitaire des Aliments* (AFSSA-France).

*Sheep-scrapie* isolate SC-UCD-99 was obtained from the brain of an Irish ARQ/ARQ sheep naturally infected with scrapie (provided by the Veterinary Research Laboratory, Abbotstown, Ireland).

*Mouse-RML scrapie* was obtained from the brain of terminally diseased C57/Bl6 mice inoculated with Rocky Mountain Laboratory (RML) scrapie.

*Human-sCJD* (met 129) isolate (Ref.: NHBX0/0001) was obtained from the National Institute for Biological Standards and Control, Potters Bar, United Kingdom.

All inocula were prepared from brain tissues as 10% (w/v) homogenates in 5% glucose. To minimize the risk of bacterial infection, inocula were preheated for 10 min at 70°C before inoculation in mice.

### Mouse transmission studies

All the isolates were inoculated in three different mouse models: i) BoPrP-Tg110 transgenic mouse line expressing bovine PrP^C^ at a 8-fold level [24], [26], [28], ii) PoPrP-Tg001 mouse line expressing porcine PrP^C^ at a 4-fold level [24], [26], [28], and iii) HuPrP-Tg340 mouse line expressing human PrP^C^ at a 4-fold level [24], [26], [28]; all of them express the transgenic PrP on a mouse PrP null background.

Individually identified 6–10 weeks-old mice were anesthetized with isoflurane and inoculated with 2 mg of brain homogenate in the right parietal lobe using a 25-gauge disposable hypodermic needle. Mice were examined twice weekly for neurological signs of prion disease and were euthanized by cervical dislocation when progression of the disease was evident or at the end of the study (650–700 dpi). The animals were humanely euthanized once a definitive diagnosis had been made or earlier if showing signs of distress or loss of up to 20% of body weight. A mouse was considered positive for prion disease when it showed two or three of 10 signs of neurological dysfunction previously described [30], [31]. Definitive diagnosis was made on observation of one confirmatory sign of prion disease and animals were culled at this point. Confirmatory signs included ataxia, generalized tremor, loss of righting reflex, limb paralysis, extensive pilo-erection and sustained hunched posture.

Once euthanized, a necropsy was performed and the brain was taken. A part of the brain was fixed by immersion in neutral-buffered 10% formalin (4% formaldehyde) to quantify spongiform degeneration by histopathology and PK resistant PrP accumulation (PrP^res^) by immunohistochemistry (IHC) or histoblotting and the rest was frozen at −20°C before determining the presence of PrP^res^ by Western blot (WB). In all cases, survival time and attack rate were calculated for each isolate. Survival time was expressed as the mean of the survival days post inoculation (dpi) of all the mice scored positive for PrP^res^, with its correspondent standard error. Attack rate was determined as the proportion of mice scored positive for PrP^res^ from all the mice inoculated. Brain homogenates from PrP^res^ positive mice, when available, were used for further passaging. When all mice were scored negative for PrP^res^ on primary passage, PrP^res^-negative brain homogenates were used for second passage.

### Western blot analysis of PrP^res^

175±20 mg of frozen brain tissue were homogenized in 5% glucose in distilled water in grinding tubes (Bio-Rad) adjusted to 10% (w/v) using a TeSeE Precess 48 homogenizer (Bio-Rad) following the manufacturer's instructions. The presence of PrP^res^ was determined by Western blot, following the procedure described below and using the reagents of the ELISA commercial test (TeSeE, Bio-Rad). 10–100 µl of a 10% (w/v) brain homogenate were diluted in a 10% (w/v) negative sheep brain homogenate, to obtain a 200 µl final volume. Homogenates were incubated for 10 min at 37°C with 200 µl of a 2% proteinase K solution (in buffer A). PrP^res^ was recovered as a pellet after addition of 200 µl of buffer B and centrifugation at 15,000× *g* for 7 min at 20°C. Supernatants were discarded and pellets were dried inverted over absorbent paper for 5 min. Pellets were solubilised in Laemmli buffer and samples were incubated for 5 min at room temperature, solubilised, and heated at 100°C for 5 min. Samples were centrifuged at 20,000× *g* for 15 min at 20°C and supernatants were recovered and loaded on a 12% Bis-Tris Gel (Criterion XT, BioRad or NuPage, Invitrogen). Proteins were electrophoretically transferred onto PVDF or nitrocellulose membranes (Millipore). Membranes were blocked O/N with 2% BSA blocking buffer. For immunoblotting, monoclonal antibodies Sha31 [32] and 12B2 [33] were used at a concentration of 1 µg/mL. Sha31 recognizes the _156_YEDRYYRE_163_ epitope of the bovine-PrP sequence while 12B2 recognizes the _101_WGQGG_105_ epitope of the bovine-PrP sequence. Immunocomplexes were detected by incubating the membranes for 1 hour with horseradish peroxidase conjugated anti mouse IgG (Amersham Pharmacia Biotech). Immunoblots were developed with enhanced chemiluminescence using ECL Plus reagent (GE Healthcare Amersham Biosciences).

### PrP^Sc^ proteinase K resistance ELISA test

PrP^Sc^ detection was carried out using two sandwich ELISA tests (TeSeE, Bio-Rad) following the manufacturer's instructions. The assay protocol includes a purification of PrP^Sc^ (TeSeE purification kit) consisting of (i) digestion of PrP^C^ with PK, (ii) precipitation of PrP^Sc^ by centrifugation and (iii) denaturation of PrP^Sc^ at 100°C, before immuno-enzymatic detection. In this ELISA, the capture antibody recognizes the octarepeat region of PrP [34], while the detection antibody binds to the core part of the protein [32].

PK resistance of the PrP^Sc^ portion recognized in the ELISA test was determined by measurement of the ELISA specific signal recovered after digestion with different concentrations of PK in ‘buffer A’ reagent (TeSeE purification kit, Bio-Rad). Each sample was first diluted in normal brain homogenate (between 100 and 10 000 fold) until a signal between 1.5 and 2 absorbance units was obtained after digestion with 50 µg/ml of PK. Triplicates of equilibrated samples were then submitted to a PK digestion with concentrations ranging from 50 to 300 µg/ml, before PrP^Sc^ precipitation and ELISA detection. Results were expressed as the percentage of residual signal as compared with the signal after 50 µg/ml PK digestion (lowest PK concentration). In each assay, two standardized controls (scrapie and BSE from sheep) were used as an internal standard.

### Histopathology

All analyses of mouse brains were performed as previously described [35]. Briefly, samples were fixed in neutral-buffered 10% formalin (4% formaldehyde) before paraffin embedding. After deparaffinization, 2-µm-thick tissue sections were stained with haematoxylin and eosin. Lesion profiles of the brains were established according to the standard method described by Fraser and Dickinson [36]. For paraffin-embedded tissue (PET) blots, the protocol described by Andréoletti et al. [37] was used.

### Statistical analysis

The PK resistance profiles were compared by Wilcoxon matched-pairs signed-rank test. Two-tailed P-values <0.05 were considered statistically significant.

Nonparametric Mann-Whitney-U test was applied to establish statistically significant differences in survival times of the different mouse models inoculated with the different BSE-derived prions. A difference of P<0.05 was considered significant. Statistical analysis was performed using PAST software (PAlaeontological STatistics, version 1.81).

## Results

In this work we investigated the biochemical and biological characteristics of a panel of BSE-derived prions corresponding to BSE passaged in both natural host species (cattle, sheep, pigs, mice and humans) and in transgenic mice overexpressing the corresponding PrP^C^ amino acid sequences. These BSE-derived prions have been characterized in comparison with the original cattle-BSE prions, as well as with other non-BSE related prion strains as controls. Human-vCJD was included as another BSE-derived prion [13], [14] while atypical cattle-BSE H, sheep-scrapie, mouse-RML and human-sCJD prions were included as non-BSE related prion strains.

### Biochemical features of BSE-derived prions

**Figure 1:**
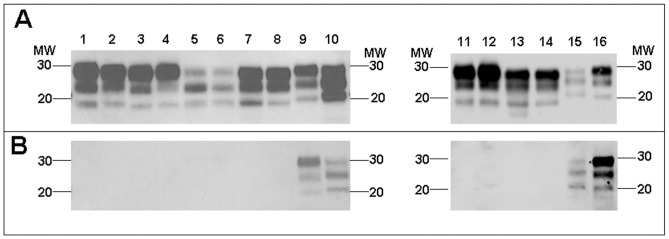
Electrophoretic profiles and antibody labelling of PrP^res^ in BSE-derived prions. PrP^res^ was detected by western blot using the mAbs Sha31 (A) and 12B2 (B) in the different BSE-derived prions propagated either in natural hosts or in transgenic mouse models over-expressing their corresponding PrP^C^ sequence: cattle-BSE (lanes 1 and 11), BoTg-BSE (lanes 2 and 12), sheep-BSE (lane 3), OvTg-BSE (lane 4), pig-BSE (lane 5), PoTg-BSE (lane 6), mouse-BSE (lane 7), MoTga20-BSE (lane 8), human-vCJD (lane 13), HuTg-BSE (lane 14). Sheep-scrapie (lane 9), mouse-RML (lane 10), human-sCJD (lane 15) and atypical cattle-BSE H (lane 16) were included as control non-BSE related prions. Panels A and B were loaded with the same quantities of PrP^res^ extracted from each sample. MW, molecular weight in kilodaltons.

**Figure 2:**
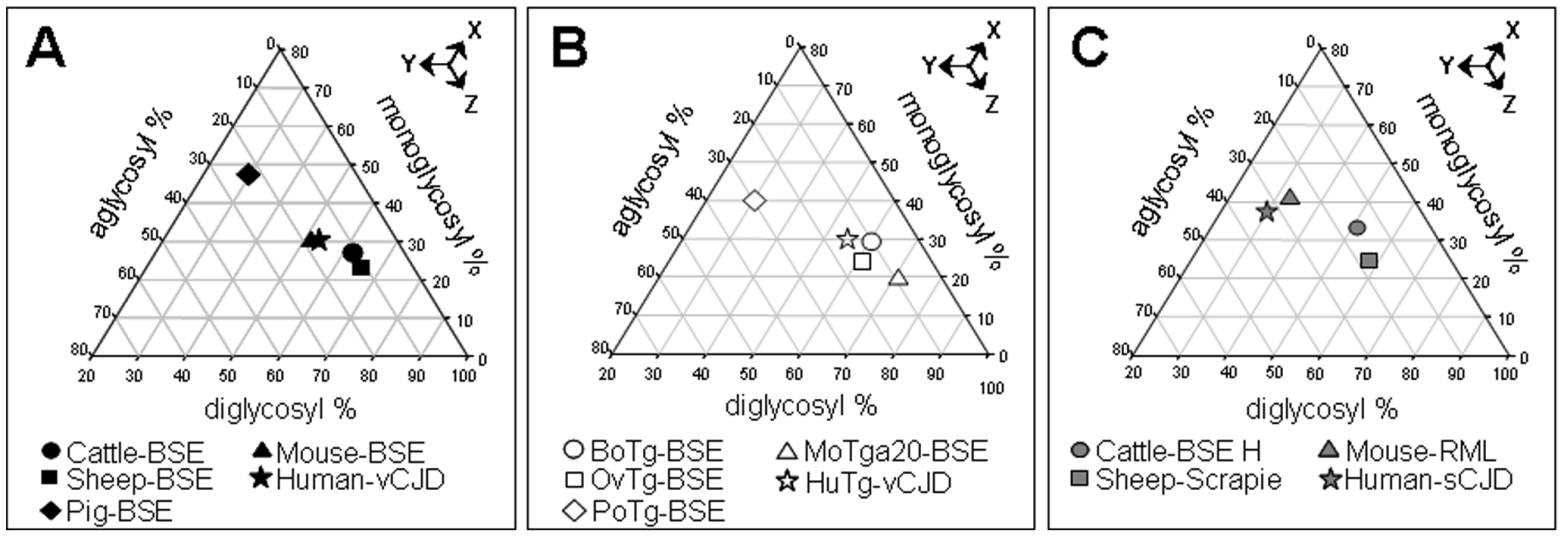
Glycoform ratios of PrP^res^ detected by Western blotting. PrP^res^ was detected with the Sha31 monoclonal antibody in the different BSE-derived prions propagated either in the natural hosts (A) or in transgenic mouse models over-expressing their corresponding PrP^C^ sequence (B). Atypical cattle-BSE H, sheep-scrapie, mouse-RML and human-sCJD isolates (C) are included for comparison purposes. Values are the normalized means from at least six repeated runs. Arrows indicate the reading direction of the axis.

The Western blot pattern of BSE-derived PrP^res^ was analysed using Sha31 mAb (Figure 1A) binding to the YEDRYYRE epitope (amino acids 148–155 of sheep PrP) which is conserved in the PrP of all the species analysed here. No major differences were found in the PrP^res^ electromobility of cattle-BSE compared to the different BSE-derived prion isolates (Figure 1A). In all cases, BSE-derived prion isolates propagated in the different hosts, and showed a faster electromobility than the atypical cattle-BSE H, sheep-scrapie, mouse-RML and human-sCJD isolates used as controls (Figure 1A). A certain variability of the PrP^res^ glycoforms was observed in the different BSE-derived prion isolates (Figure 2). BoTg-BSE, OvTg-BSE, MoTga20-BSE and HuTg-BSE showed a profile dominated by a diglycosylated PrP^res^ fraction (>50%) and a weak unglycolysated PrP^res^ fraction (<20%). In contrast, PoTg-BSE showed a profile dominated by the monoglycosylated PrP^res^ fraction (∼40%). In all cases, the glycoprofiles observed in the natural hosts were similar to those observed in the corresponding transgenic mouse models.

12B2 antibody is specific for the PrP amino acid sequence 89–93 that is located N-terminally of the BSE main PK cleavage site [33] and conserved in all the species tested here. PrP^res^ from atypical cattle-BSE H, sheep-scrapie, mouse-RML and human-sCJD isolates was recognized equally well by both Sha31 and 12B2 mAbs. Conversely, PrP^res^ from BSE in all investigated hosts was not recognized by 12B2 mAb (Figure 1B). This observation indicates that after propagation in the different animal models, the 12B2 epitope remains unprotected against PK digestion, as it is in the original cattle-BSE inoculum. After propagation in cattle, pigs, sheep, conventional mice and humans the entire PrP^res^ Western blot features of BSE, including electromobility, glycoprofile and affinity to 12B2 mAb were similar to those observed in Tg mouse models over-expressing the same PrP^C^ sequences (Figures 1 and 2).

**Figure 3:**
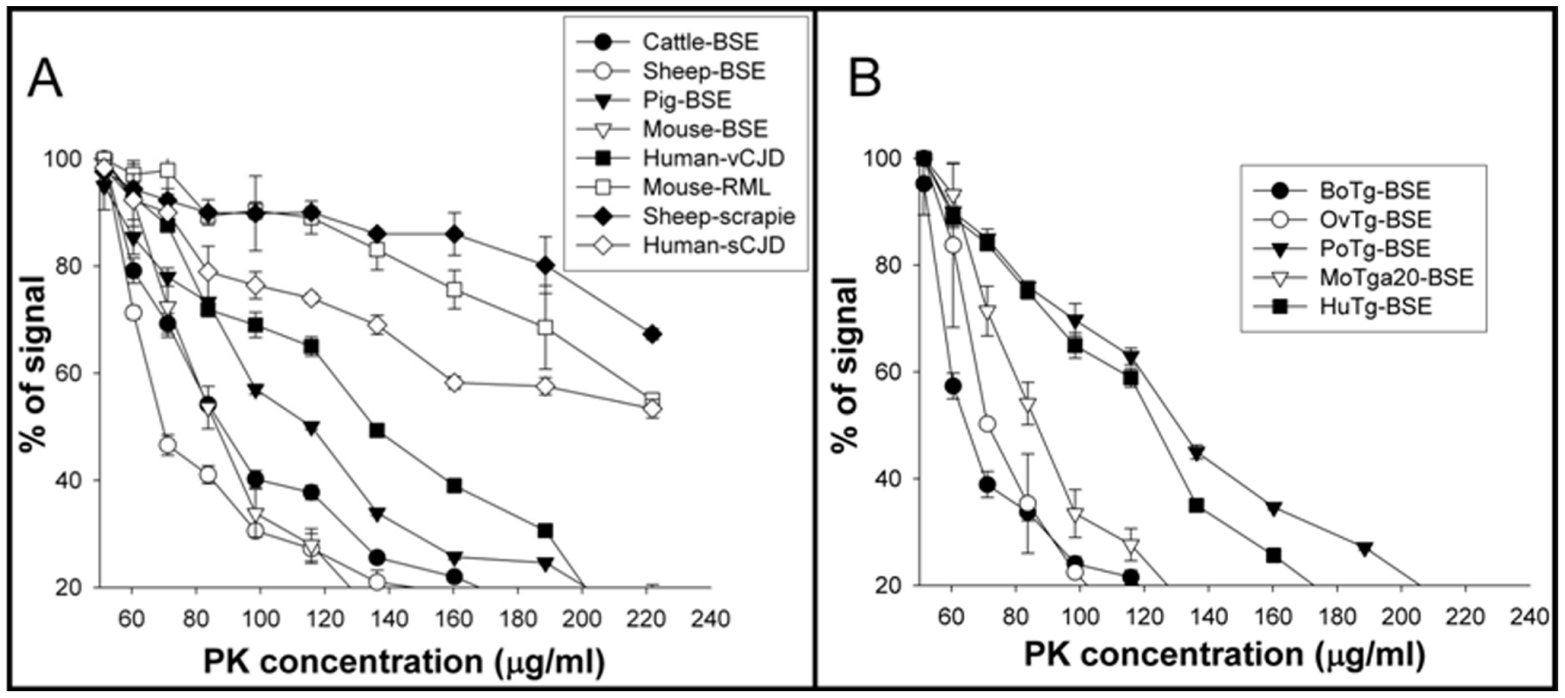
PrP^Sc^ proteinase K resistance ELISA test in BSE-derived prions. A) BSE-derived prions propagated in several host species (cattle-BSE, sheep-BSE, pig-BSE, mouse-BSE and human-vCJD); mouse-RML, sheep-scrapie and human-sCJD isolates are included for comparison purposes. B) BSE-derived prions propagated in transgenic mice expressing PrP^C^ of these species (BoTg-BSE, OvTg-BSE, PoTg-BSE, MoTga20-BSE and HuTg-BSE).

PrP^Sc^ PK resistance ELISA was recently described as a tool for biochemical classification of s-CJD cases [38]. The assay is based on the characterization of the dynamics of the N-terminal PK cleavage of the PrP^Sc^ under increasing PK concentration. Using this method, BSE propagated in hosts expressing bovine, ovine and murine PrP displayed a similarly low PK resistance profile (Figure 3). In contrast, BSE in hosts expressing porcine or human PrP showed a significantly higher PK resistance profile than the original cattle-BSE inoculum; but remained less resistant to PK digestion than the non BSE-related prions (sheep-scrapie, mouse-RML or human-sCJD) isolates used as controls (Figure 3). Here again, the PK resistance profiles observed in the conventional host species (Figure 3A) were similar to those observed in the corresponding transgenic mouse models (Figure 3B).

### Transmission features of BSE-derived prions

The biological properties of BSE after propagation in different host species were assessed through passage in three different mouse models (BoPrP-Tg110, PoPrP-Tg001 and HuPrP-Tg340) expressing bovine, porcine or human PrP, respectively [24], [26], [28]. For most of the tested isolates, 2–3 passages have already been completed in these mouse models (Table 1).

**Table 1:** Transmission features of BSE-derived prions in comparison with non-BSE related prions in different mouse models (BoPrP-Tg110, PoPrP-Tg001 and HuPrP-Tg340).

Inocula	PrP^Sc^ sequence	Mean survival time in days ± sem (n/n_0_)^a^							
		BoPrP-tg110 mice			PoPrP-tg001 mice			HuPrP-tg340 mice	
		1^st^ passage	2^nd^ passage	3^rd^ passage	1^st^ passage	2^nd^ passage	3^rd^ passage	1^st^ passage	2^nd^ passage
**Cattle-BSE**	**Bovine**	**295±12 (6/6)**	**265±35 (6/6)**	**243±7 (6/6)**	650 (1/6)	199±7 (5/5)	173±13 (5/5)	700 (1/8)	633±32 (4/4)
**BoTg-BSE**	**Bovine**	**265±35 (6/6)**	**243±7 (6/6)**	**ND**	372 (1/6)	208±12 (6/6)	ND	>700 (0/5)	644±87 (3/6)
**Cattle-BSE H**	**Bovine**	**292±11 (7/7)**	**282±05 (6/6)**	**ND**	>650 (0/6)	>650 (0/6)	ND	>700 (0/6)	>700 (0/6)
**Sheep-BSE**	**Ovine**	229 ±11 (7/7)	237±5 (6/6)	245±15 (5/5)	418±28 (6/6)	165±6 (6/6)	169±8 (6/6)	690±83 (6/6)	564±39 (5/5)
**OvTg-BSE**	**Ovine**	223±10 (5/5)	238±17 (6/6)	236±9 (6/6)	384±87 (6/6)	ND	ND	604±30 (5/5)	ND
**Sheep-scrapie**	**Ovine**	560±26 (7/9)	289±15 (6/6)	285±9 (6/6)	>650 (0/7)	>650 (0/5)	ND	>700 (0/6)	>700 (0/6)
**Pig-BSE**	**Porcine**	323±17 (6/6)	246±43 (6/6)	ND	**183±8 (6/6)**	**ND**	**ND**	684±115 (2/4)	702±25 (5/5)
**PoTg-BSE**	**Porcine**	329±49 (6/6)	251±20 (6/6)	239±5 (6/6)	**187±4 (5/5)**	**190±10 (7/7)**	**178±9 (6/6)**	675±33 (3/6)	550±13 (5/5)
**Mouse-BSE**	**Murine**	366±34 (5/5)	310±36 (6/6)	249±3 (6/6)	650 (1/6)	201±12 (6/6)	ND	700 (1/5)	ND
**MoTga20-BSE**	**Murine**	358±32 (6/6)	282±23 (6/6)	252±23 (6/6)	506 (1/6)	ND	ND	615±88 (3/6)	ND
**Mouse-RML**	**Murine**	>650 (1/6)	309±11 (7/7)	264±11 (4/4)	>650 (0/6)	>650 (0/6)	ND	>700 (0/5)	ND
**Human-vCJD**	**Human**	334±64 (6/6)	244±2 (5/5)	ND	556±81 (6/6)	212±6 (6/6)	172±9 (5/5)	**626±29 (6/6)**	**612±69 (6/6)**
**HuTg-BSE**	**Human**	318±13 (5/5)	265±9 (5/5)	250±32 (6/6)	486±31 (5/7)	ND	ND	**690±5 (4/4)**	**522±87 (5/5)**
**Human-sCJD**	**Human**	>650 (0/6)	ND	ND	>650 (0/6)	>650 (0/6)	ND	**214±6 (5/5)**	**198±7 (6/6)**

a Intracerebral inoculation with 2 mg brain tissue equivalent; n/n_0_: diseased, PrP^res^ positive/inoculated animals; SEM: standard error of the mean.

ND: not determined.

Results from homologous transmissions (host and donor share the same species-PrP sequence) are marked in bold.

In BoPrP-Tg110 mice, the different BSE-derived prions were fully transmitted (with a 100% attack rate) in primary passage, irrespective of their producing host (Table 1). Either no modification or low reduction of the survival time was observed between the first and the second passage. Although in some cases this reduction was significant, as in Pig-BSE and Hu-vCJD, it was always lower than 25%. These results are consistent with a lack of (or a weak) transmission barrier when the different BSE-derived prions are transmitted to BoPrP-Tg110 mice.

In both PoPrP-Tg001 and HuPrP-Tg340 mouse models, all BSE-derived prions were able to be transmitted although a significant species barrier was observed in most of them when inoculated in mice with a heterologous PrP^C^. For BSE prions propagated in bovine, porcine, murine or human-PrP hosts, the transmission efficiency in both mouse models was similar to that observed for the cattle-BSE isolate. However, the OvTg-BSE isolate showed higher transmission efficiency on primary passage (higher attack rate and/or reduced survival times), as compared to the cattle-BSE isolate, in the three mouse models used. Similar increased transmission efficiency was previously reported for sheep-BSE in these mouse models [22]–[24], and it was confirmed herein with a new Sheep-BSE isolate (Table 1). Strikingly, the transmission efficiency observed in the three mouse models inoculated with BSE propagated in natural hosts (cattle, sheep, pigs, mice and humans) was similar to that obtained with the BSE agent propagated in the transgenic mouse models overexpressing the same PrP^C^ sequences (Table 1). It is interesting to note that, after 2–3 passages in the same mouse model, all BSE-derived prions showed similar survival times, indicating no stable modifications of the biological properties of the BSE agent during trans-species transmission.

A different scenario was observed when non-BSE related prions were inoculated into the same three mouse models. While all BSE-derived prions were transmitted with a 100% attack rate irrespective of their producing host, the sheep-scrapie SC-UCD-99 and the mouse-RML isolates showed prolonged incubation times and reduced attack rates on primary passage to BoPrP-Tg110 mice; and the human-sCJD isolate was not transmitted to BoPrP-Tg110 (Table 1). Moreover, none of the non BSE-derived prions were transmitted to the PoPrP-Tg001 mouse model and only the human-sCJD was transmitted to the HuPrP-Tg340 mouse model (Table 1). Because these same three inocula displayed short and stable incubation periods, without any reduction between the first and second passages in transgenic mice expressing an homologous PrP^C^ (data not shown), a low infectivity titer for these isolates cannot be considered to be the cause of their impaired transmission.

**Figure 4:**
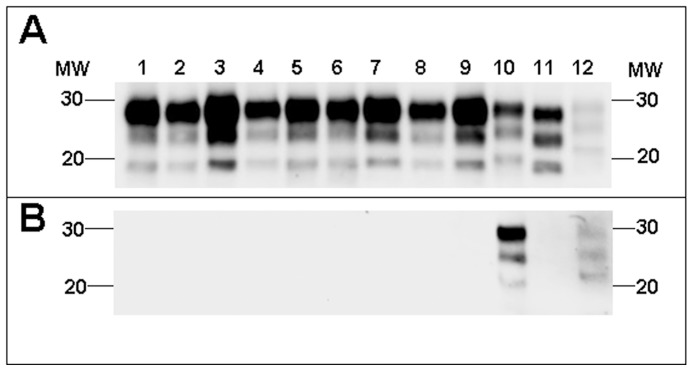
PrP^res^ in BoPrP-Tg110 mice. Electrophoretic profiles and antibody labelling of PrP^res^ as detected by mAbs Sha31 (A) and 12B2 (B) in brain extracts from BoPrP-Tg110 mice inoculated with the different BSE-derived prions: cattle-BSE (lane 1), sheep-BSE (lane 2), OvTg-BSE (lane 3), pig-BSE (lane 4), PoTg-BSE (lane 5), mouse-BSE (lane 6), MoTga20-BSE (lane 7), human-vCJD (lane 8), HuTg-BSE (lane 9). Brain extracts from BoPrP-Tg110 mice inoculated with atypical cattle-BSE H (lane 10), sheep-scrapie (lane 11) and mouse-RML (lane 12) were included as a control non-BSE related prion propagated in the same mouse model. Panels A and B were loaded with the same quantities of PrP^res^ extracted from each sample. MW, molecular weight in kilodaltons.

The brains of BoPrP-Tg110 mice inoculated with the different BSE-derived prions showed similar PrP^res^ biochemical signatures (Figure 4, lanes 1–9) as no significant differences could be observed in terms of electrophoretic mobility, glycoforms ratio and lack of reactivity to 12B2 mAb (Figure 4, lanes 1–9). This PrP^res^ biochemical signature was completely different to that observed in mouse brains inoculated with the atypical cattle-BSE H, sheep-scrapie or mouse-RML isolates (Figure 4, lanes 10–12). These differential characteristics were also maintained after a second passage in these mice (data not shown).

**Figure 5:**
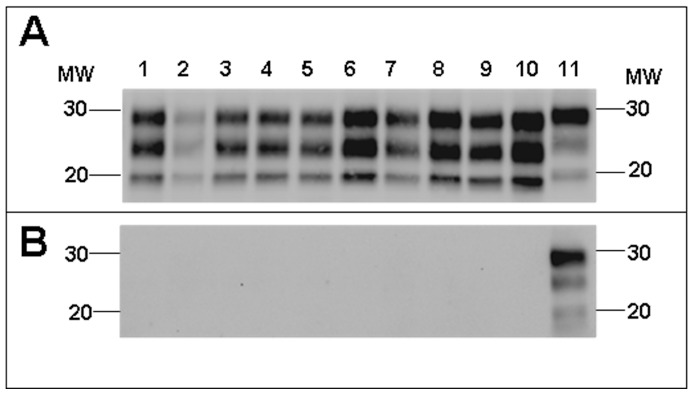
PrP^res^ in PoPrP-Tg001 mice. Electrophoretic profiles and antibody labelling of PrP^res^ as detected by mAbs Sha31 (A) and 12B2 (B) in brain extracts from PoPrP-Tg001mice inoculated with the different BSE-derived prions: cattle-BSE (lane 1), BoTg-BSE (lane2), sheep-BSE (lane 3), OvTg-BSE (lane 4), porcine-BSE (lane 5), PoTg-BSE (lane 6), mouse-BSE (lane 7), MoTga20-BSE (lane 8), human-vCJD (lane 9), HuTg-BSE (lane 10). Atypical cattle-BSE H isolate (lane 11) was included as positive control for 12B2 antibody labelling. Panels A and B were loaded with the same quantities of PrP^res^ extracted from each sample. MW, molecular weight in kilodaltons.

**Figure 6:**
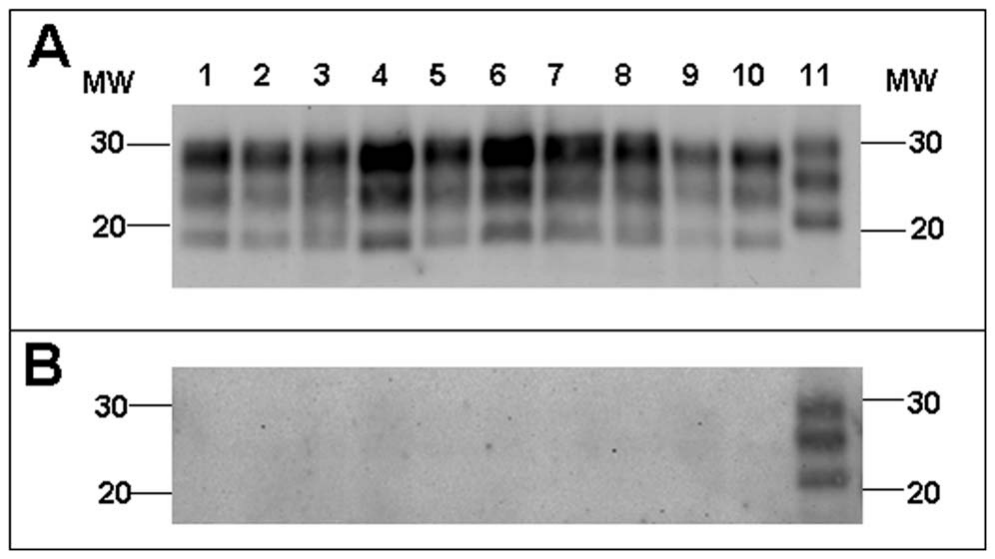
PrP^res^ in HuPrP-Tg340 mice. Electrophoretic profiles and antibody labelling of PrP^res^ as detected by mAbs Sha31 (A) and 12B2 (B) in brain extracts from HuPrP-Tg340 mice inoculated with the different BSE-derived prions: cattle-BSE (lane 1), BoTg-BSE (lane2), sheep-BSE (lane 3), OvTg-BSE (lane 4), pig-BSE (lane 5), PoTg-BSE (lane 6), mouse-BSE (lane 7), MoTga20-BSE (lane 8), human-vCJD (lane 9), HuTg-BSE (lane 10). Brain extract from HuPrP-Tg340 mice inoculated with human-sCJD isolate (lane 11) was included as a control non-BSE related prion propagated in the same mouse model. Panels A and B were loaded with the same quantities of PrP^res^ extracted from each samples. MW, molecular weight in kilodaltons.

In the PoPrP-Tg001 mouse model, all BSE-derived prions showed the same and unique PrP^res^ biochemical signature (PrP^res^ electrophoretic mobility, glycoprofile and lack of reactivity to 12B2 mAb) in the brains of infected mice (Figure 5), while none of the non-BSE related prion isolates was able to infect these mice and no PrP^res^ was detected in their brains when sacrificed at 650 dpi. Similarly, all BSE-derived prions showed identical PrP^res^ biochemical signatures in HuPrP-Tg340 mice (Figure 6) which was completely different to that of human-sCJD, the only non-BSE related prion isolate able to infect this mouse model.

**Figure 7:**
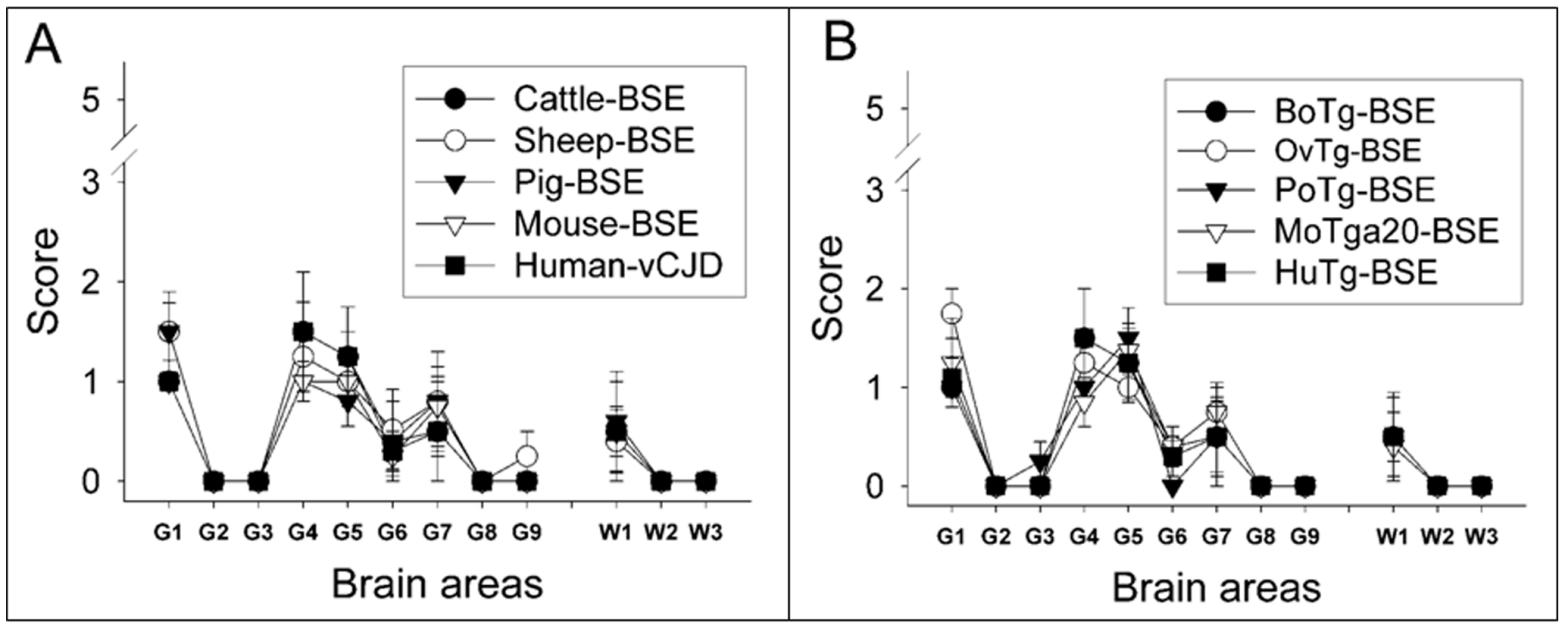
Lesion profile in the brain of BoPrP-Tg110 mice. A) Lesion profile in BoPrP-Tg110 mice infected (1^st^ passage) with the different BSE-derived prions propagated either in the natural hosts (A) or in transgenic mouse models expressing their corresponding PrP^C^ sequence (B). Lesion scoring is determined for nine areas of grey matter (G) and white matter (W) in the mouse brains: dorsal medulla (G1), cerebellar cortex (G2), superior colliculus (G3), hypothalamus (G4) medial thalamus (G5), hippocampus (G6), septum (G7), medial cerebral cortex at the level of the thalamus (G8) and at the level of the septum (G9), cerebellum (W1), mesencephalic tegmentum (W2) and pyramidal tract (W3).

We also compared the neuropathological phenotypes of all BSE-derived agents by histopathological examination and PrP^res^ histoblotting. Lesion profiles of BoPrP-Tg110 mice inoculated with BSE prions derived from different natural and transgenic hosts were all similar to that obtained with the original cattle-BSE isolate (Figure 7) but different to those previously described in these mice inoculated with non-BSE related isolates as atypical BSE-H [39] or sheep-scrapie [22]. The PrP^res^ deposition patterns of all BSE-derived prions were similar to that previously reported for cattle-BSE in these BoPrP-Tg110 mice, but different to that observed with H-type BSE when inoculated in the same mouse model [39].

Similarly, in PoPrP-Tg001 we found a similar and unique neuropathological phenotype for all BSE-derived agents irrespective of their producing host. In all cases, the vacuolation profile and the PET blot PrP^res^ deposition pattern (data not shown) were indistinguishable from those previously reported for both cattle-BSE and sheep-BSE isolates in this porcine-PrP mouse model [23]. Moreover, in HuPrP-Tg340 mice all BSE-derived prions also showed similar neuropathological features (data not shown) which were identical to those previously observed in the brains of those mice infected with either human-vCJD or cattle-BSE [24].

## Discussion

Once the species barrier is crossed, both biochemical and biological properties of prion strains can change [6] as an adaptation to the new species. This suggests that when the barrier is crossed, some features of the newly generated prion could be related to the original prion strain, while others could be linked to the new host PrP^C^ amino acid sequence or to other host factors. Through the biochemical and biological characterization of the BSE-derived prions tested herein (sharing the same original strain but with different PrP^Sc^ amino acid sequences) in comparison to other non-BSE related prions sharing the same PrP^Sc^ amino acid sequences, we can try to determine the role of these elements (strain-conformation, PrP^Sc^ primary sequence and other species-specific host factors) in either the species barrier phenomenon or the species barrier passage consequences.

### Alterations of PrP^Sc^ biochemical properties after BSE trans-species transmission

Most of the biochemical analyses applied, including the PK resistance assay, which was recently described as a tool for describing intra-species PrP^Sc^ variability [38], [40], revealed subtle differences in PrP^Sc^ depending on the host PrP sequence in which the BSE agent was propagated. The only biochemical feature that was apparently conserved in all BSE-derived prions after passage in the different species was the lack of reactivity of PrP^res^ to the N-terminal binding antibody 12B2 (Figure 1), indicating that the characterization of the abnormal PrP N-terminal truncation by PK could remain a robust approach for potential BSE agent identification when screening TSE isolates from different species.

Similar changes of the abnormal PrP^Sc^ biochemical signature were observed when BSE was propagated either in transgenic mouse models or in the corresponding natural host (cattle, sheep, pigs, conventional mice and humans). These results support the statement that the alteration of the PrP^Sc^ biochemical features induced by trans-species transmission is only dependent on the host PrP^C^ amino acid sequence and not influenced by the PrP^C^ expression level or other host genetic factors.

### Prion transmission barrier is mainly modulated by strain properties rather than by the PrP^Sc^ amino acid sequence of the inoculum

A transmission barrier could have been expected when transmitting BSE propagated in different species back to bovine-PrP expressing mice. However, our results in BoPrP-Tg110 mice (Table 1) indicate that such BSE-derived prions were transmitted with no or a very weak transmission barrier irrespective of their PrP^Sc^ sequence. In contrast, a clear transmission barrier was observed with non-BSE related isolates propagated in hosts with an identical PrP^C^ sequence. Moreover, these prion isolates, sharing identical PrP^Sc^ but from different strains, also showed a different transmission ability in the other two mouse models (PoPrP-Tg001 and HuPrP-Tg340) tested herein (Table 1). Our results demonstrated that there is no clear relationship between the degree of identity of the PrP of the donor and recipient species and susceptibility, consistent with the view that the prion strain provides a major contribution to the species barrier. In other words, the prion species barrier is modulated by strain properties (presumably conformation-dependent) rather than by PrP amino acid sequence differences between host and donor.

### BSE-derived agents can increase their transmission efficiency to others species depending on the PrP^Sc^ sequence

After re-inoculation into transgenic mice expressing bovine, porcine or human PrP, BSE-derived agents displayed characteristics similar to the original cattle-BSE agent in the same models in terms of the biochemical PrP^Sc^ profile, lesion profile and abnormal PrP distribution in the brain, which are the main criteria currently used for prion strain clustering. Despite all the BSE-derived prions apparently belonging to the ‘BSE cluster’, some differences in transmission efficiency could be observed. Indeed, the passage of BSE first in a host with an ovine-PrP (sheep-BSE and OvTg-BSE) increased its transmission efficiency in the three mouse models tested herein (Table 1), whereas a passage in hosts harboring porcine, murine or human-PrP resulted in a transmission efficiency similar to that observed with the original cattle-BSE agent (Table 1). Although a higher titre of the BSE agent in hosts expressing ovine-PrP cannot be excluded, these differences could be attributed to an effect of host-donor PrP sequence interaction as previously proposed [24]. Apparently, an ovine-PrP^BSE^ sequence facilitates the conversion of others species-PrP^C^ (human, porcine and even bovine) compared to other species-PrP^BSE^ including the bovine, murine or porcine one. Nevertheless, the PrP^Sc^ primary sequence influence seems to depend strongly on the strain involved, since the sheep-scrapie isolate was not transmitted to HuPrP-Tg340 or PoPrP-Tg001 mice and it was poorly transmitted to BoPrP-Tg110 mice (Table 1). These results support the hypothesis that the trans-species transmission of BSE agent can alter its transmission efficiency to others hosts without fundamental alteration of other strain specific features. Since similar results were observed when BSE was propagated either in natural hosts or mice overexpressing the same PrP^C^ sequences, such alteration has to be considered independent of both the PrP^C^ expression level and genetic factors other than the PrP^C^ sequence.

Taken together all these findings unambiguously demonstrate that the species barrier and its passage consequences are uniquely driven by the PrP^C^ sequence, and not by other host genetic factors, demonstrating the validity of transgenic PrP animals as models for studies of the species barrier.

The capacity of BSE-derived agents to maintain (or even increase) their transmission efficiency to several species could impact on the trans-species transmission risk under natural exposure conditions. This should always be kept in mind for risk assessment of the potential spread of non-bovine BSE in field cases, since there is a risk that species other than cows with no, or a low, transmission barrier will propagate BSE infection in several other species, including humans.
